# 11,12-Diacetyl-carnosol Protects SH-SY5Y Cells from Hydrogen Peroxide Damage through the Nrf2/HO-1 Pathway

**DOI:** 10.1155/2022/4376812

**Published:** 2022-05-17

**Authors:** Qingyi Luo, Weiyan Hu, Haofei Yu, Rongping Zhang, Xinglong Chen

**Affiliations:** ^1^Department of Imaging, Yanan Hospital of Kunming City, The Affiliated Hospital of Kunming Medical University, Kunming 650051, Yunnan Province, China; ^2^Yunnan Province Key Laboratory of Cardiovascular Diseases, Kunming 650051, Yunnan Province, China; ^3^School of Pharmaceutical Science & Yunnan Key Laboratory of Pharmacology for Natural Products, Kunming Medical University, Kunming 650500, China; ^4^College of Chinese Materia Medica and Yunnan Key Laboratory of Southern Medicine Utilization, Yunnan University of Chinese Medicine, Kunming 650500, China

## Abstract

**Background:**

Oxidative stress-induced neurotoxicity plays a key role in Alzheimer's disease (AD). 11,12-Diacetyl-carnosol (NO.20), an acetylated derivative of carnosol extracted from rosemary, displays a high antioxidative effect in vitro.

**Purpose:**

We investigated the neuroprotective effect of NO.20 on H_2_O_2_-induced neurotoxicity in human neuroblastoma SH-SY5Y cells and its possible mechanism.

**Results:**

We found that NO.20 pretreatment (1 *μ*M for 1 h) had cytoprotective effects and weakened H_2_O_2_-induced damage in SH-SY5Y cells by reducing viability loss, apoptotic rate, and reactive oxygen species production. In addition, NO.20 inhibited H_2_O_2_-induced mitochondrial dysfunctions: it alleviated mitochondrial membrane potential loss and cytochrome c release, decreased the Bax/Bcl-2 ratio, and reduced caspase-3 expression. NO.20 also downregulated malondialdehyde and upregulated glutathione. Furthermore, NO.20 pretreatment caused the nuclear translocation of the transcription factor NF-E2-related factor 2 (Nrf2), increasing heme oxygenase-1 (HO-1) expression in SH-SY5Y cells. Notably, we found that silencing Nrf2 using small interfering RNA (siRNA) suppressed the NO.20-induced HO-1 expression and abolished the neuroprotective effect of NO.20.

**Conclusion:**

These results demonstrate that NO.20 protects SH-SY5Y cells from H_2_O_2_-induced neurotoxicity by activating the Nrf2/HO-1 pathway. Thus, the neuroprotective and antioxidative stress effects of NO.20 may make it a promising neuroprotective compound for AD treatment.

## 1. Introduction

Alzheimer's disease (AD), the most common cause of dementia, is a chronic age-related neurodegenerative brain disorder that often leads to the gradual loss of memory, language, and cognitive functions and causes severe emotional and behavioral abnormalities [1]. Without an efficient treatment, the number of AD patients worldwide could reach 100 million by 2050 [2]. However, no effective treatments have been discovered due to the complex factors involved in this disease's pathogenesis. The drugs used currently can only treat the symptoms of AD by improving behavioral and cognitive impairments but do little to stop its progression [3]. Therefore, new effective anti-AD drugs with minimal side effects are urgently needed.

Although AD occurrence involves multiple factors, several lines of evidence suggest that oxidative stress-induced neuronal damage and death play a crucial role in AD progression [4]. Oxidative stress involves reactive oxygen species (ROS) overproduction, which damages the structure and function of various biomolecules: ROS cleaves DNA, oxidizes proteins, peroxidizes lipids, and changes signal transductions, eventually leading to cell dysfunction and apoptosis [5]. Hydrogen peroxide (H_2_O_2_) is a major ROS produced during the redox process; it is commonly used to induce oxidative stress in cellular models [6]. Thus, discovering novel compounds that protect neurons from H_2_O_2_-induced oxidative stress could lead to AD treatments.

Nuclear factor-erythroid 2-related factor 2 (Nrf2) is an essential transcription factor regulating the expression of heme oxygenase-1 (HO-1), antioxidant enzymes, and other cytoprotective genes. Upregulating HO-1 expression protects cells against oxidative stress [7]. Therefore, researchers agree that HO-1 induction is a common feature of many neurodegenerative diseases and regard Nrf2 and HO-1 as essential targets in AD treatment.

*Rosmarinus officinalis* L. (rosemary) is a perennial herb of the Labiatae family native to Europe and the Mediterranean region; it has long been cultivated in China [8]. Rosemary exerts numerous biological effects, including anti-inflammatory, antioxidative, antiadipogenic, antiapoptotic, and neuroprotective effects [9, 10]. Thus, rosemary is a prominent source of novel drug candidates. In recent years, the interest in novel rosemary neuroprotective agents has grown [11]. Previous studies on rosemary mainly focused on its main chemical components, rosmarinic acid, carnosic acid, and carnosol and confirmed their neuroprotective effect. For example, carnosol can protect BV2 microglia and PC12 cells from H_2_O_2_-induced oxidative stress by upregulating Nrf2, which increases HO-1 expression [12].

Besides the known flavonoids, terpenoids, phenols, and others [13, 14], does rosemary contain other neuroprotective compounds? Our team has been working on the isolation and biological evaluation of rosemary components for many years, and we recently identified nine new and nineteen known compounds from its active fraction using liquid chromatography-mass spectrometry. Among them, an abietane diterpenoid (11,12-diacetyl-carnosol, NO.20) has attracted our attention. This acetylated carnosol derivative displayed a potent antioxidative effect without apparent cytotoxicity—its cell viability rate was above 80%, even higher than that of epigallocatechin gallate (EGCG) [15]. In view of this, this acetylated derivative (NO.20) with high biological activity and stability may be used as a new source for the development of new antioxidant agents for AD treatment. However, its specific molecular antioxidant mechanism is still unclear.

This study aimed to investigate the protective effects of the abietane diterpenoid compound NO.20 on H_2_O_2_-induced oxidative stress damage in SH-SY5Y cells and its possible neuroprotective mechanisms.

## 2. Materials and Methods

### 2.1. Chemicals

**Figure 1:**
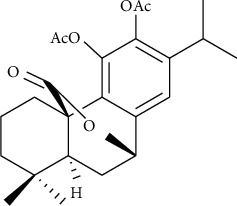
Structure of NO.20 (11,12-diacetyl-carnosol).

We extracted and separated NO.20 with a purity of more than 98% in our laboratory.  Figure 1 shows the chemical structure. Its molecular formula is C_24_H_30_O_6_ and relative molecular weight is 413.1958. We obtained the plastic materials used in cell culture from Corning, Inc. (NY, USA). We purchased H_2_O_2_ from Sigma (MO, USA), Cell Counting Kit-8 (CCK-8) from Labjic Biotechnology Company (Beijing, China), and EGCG from Meilun Biotechnology Company (Dalian, China).

### 2.2. Cell Culture

We obtained human neuroblastoma SH-SY5Y cells from the Cell Bank of the Kunming Institute of Zoology, Chinese Academy of Sciences, and cultured them in DMEM/F12 HAM (1 : 1) (Hy clone, USA) supplemented with 10% fetal bovine serum (Biological Industries, USA) and 5% penicillin/streptomycin solution (Hyclone, USA) in a humidified atmosphere containing 5% CO_2_ at 37°C.

### 2.3. CCK-8 Cell Viability Assay

We seeded the cells into 96-well plates at 5 × 10^3^ cells/well. We exposed the cells to different compounds and EGCG at 1 *μ*M and 25 *μ*M for 1 h after 24 h of subculture, added H_2_O_2_ at 300 *μ*M, and incubated for another 24 h to induce cytotoxicity and mitochondrial impairments. We then removed the culture medium, added 100 *μ*l of DMEM/F12 (1 : 1) and 10 *μ*l of CCK-8, incubated for 1 h at 37°C, and finally measured the absorbance at 450 nm using an automated microplate reader (TUR-BIO, USA). For each treatment condition, we repeated all experiments at least three times.

### 2.4. Measurement of Mitochondrial Membrane Potential

We assessed mitochondrial membrane potential (MMP) by staining SH-SY5Y cells with JC-1 and examining them through flow cytometry. We treated the cells with NO.20 and H_2_O_2_ as described above. We then incubated them in a culture medium containing 10 *μ*g/ml JC-1 (Enzo Bioscience) for 20 min at 37°C and centrifuged them for 3 min at 2000 g. We analyzed the cell samples with a flow cytometer (Rato Company, USA) after washing them with PBS. We excited JC-1 at 488 nm and measured its fluorescence intensity at 535 nm (PE-A) and 518 nm (FITC-A).

### 2.5. Cytochrome C Release Quantification

We measured cytochrome c release levels as previously reported [16].

### 2.6. ROS Quantification

We measured intracellular ROS levels using the fluorescent probe 2′,7′-dichlorofluorescein diacetate (DCFH-DA) assay kit as described previously [17].

### 2.7. NO, Glutathione (GSH), and Malondialdehyde (MDA) Quantification

We measured NO, GSH, and MDA levels in SH-SY5Y cells using commercial kits (Abcam, MA, USA) as previously reported [18, 19].

### 2.8. Quantification of Apoptotic Cells by TUNEL Staining

To quantify apoptotic cells, we prepared a TUNEL detection solution as previously reported [19].

### 2.9. Bcl-2, Bax, Caspase-3, Cytochrome C, Nrf2, and HO-1 Quantification by Western Blot Analysis

We extracted nucleoproteins and total cell proteins using a nucleocytoplasmic protein extraction kit and performed SDS-PAGE. We then transferred the proteins to polyvinylidene fluoride membranes and blocked them using 5% bovine serum albumin at room temperature for 2 h. We then added Bcl-2, Bax, caspase-3, cytochrome c, Nrf2, and HO-1 antibodies and incubated overnight at 4°C. Next, we washed the samples with TBST three times, incubated them with diluted secondary anti-rabbit IgG antibody at room temperature for 2 h, and then washed them with TBST three times before color imaging.

### 2.10. Nrf2 Knockdown by siRNA Transfection

We silenced Nrf2 by transfecting SH-SY5Y cells with Nrf2 siRNA or negative control siRNA following the manufacturer's instructions (Life Technologies, CA, USA) [20].

### 2.11. Statistical Analyses

All data were expressed as the mean ± standard deviation. We performed one-way and two-way ANOVA followed by Dunnett's multiple comparisons test using GraphPad Prism version 8.0.0 for Windows, GraphPad Software, San Diego, CA, USA, https://www.graphpad.com. In the ANOVA analyses and Student's *t*-tests, we considered that *p* < 0.05 indicated statistically significant differences between groups.

## 3. Results

### 3.1. NO.20 Prevented Cell Viability Loss and Blocked Apoptosis in SH-SY5Y Cells Exposed to H_2_O_2_

**Figure 2:**
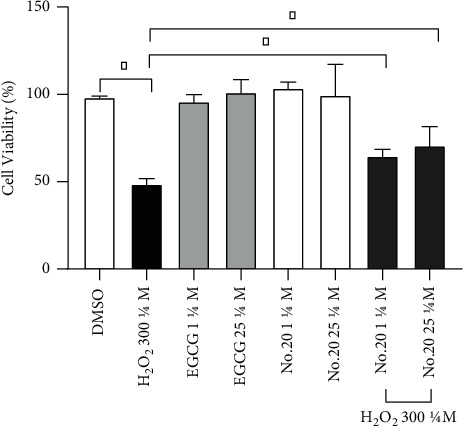
Effects of pretreatment (1 h with NO.20 or EGCG at different concentrations (1 and 25 *μ*M) on the viability of SH-SY5Y cells exposed to 300 *μ*M H2O2 for 24 h The results are presented as the mean ± SEM and represent three independent experiments. One-way ANOVA followed by Dunnett's test. ^*∗*^*P* < 0.05.

In this study, we treated the SH-SY5Y cells with 300 *μ*M of H_2_O_2_ for 24 h, as we previously determined that this dose reduced their viability by about 50% [15]. We then assessed the protective effects of NO.20 on H_2_O_2_-treated SH-SY5Y cells using the CCK-8 assay. As shown in Figure 2, NO.20 pretreatment for 1 h at 1 *μ*M and 25 *μ*M before H_2_O_2_ exposition (300 *μ*M) efficiently prevented viability loss, in a dose-dependent manner, while H_2_O_2_ alone significantly decreased cell viability (*p* < 0.05). NO.20 alone (at either concentration) did not affect the viability of SH-SY5Y cells. NO.20 showed a neuroprotective effect and no cytotoxicity. Its antioxidative effect was higher than that of EGCG (positive control) [15]. Therefore, we used 1 *μ*M as the NO.20 concentration in the subsequent experiments.

**Figure 3:**
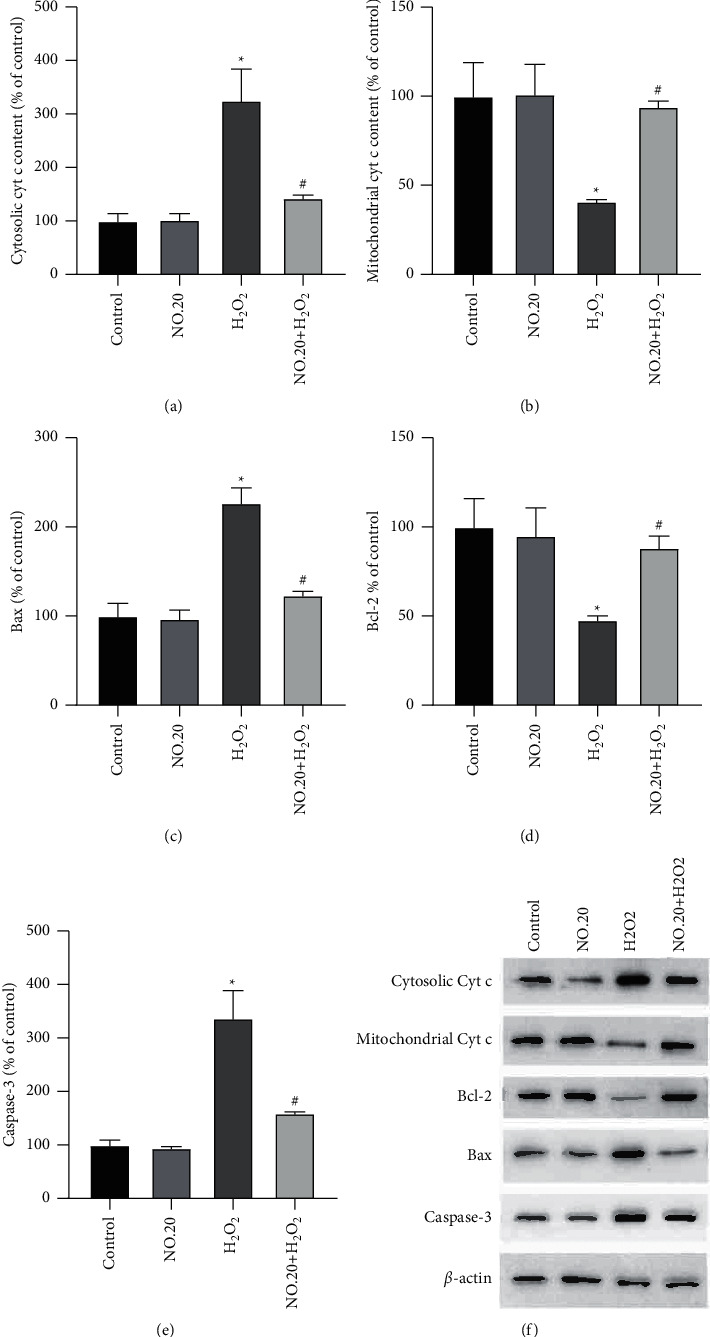
Antiapoptosis effects of NO.20 pretreatment on SH-SY5Y cells. (a) Cytosolic cytochrome c levels. (b) Mitochondrial cytochrome c levels. (c) Bax levels. (d) Bcl-2 levels. (e) Caspase-3 activity. The results are presented as the mean ± SEM and represent three independent experiments. One-way ANOVA followed by Dunnett's test. ^*∗*^*P* < 0.05 vs. the DMSO group. ^#^*P* < 0.05 vs. the H_2_O_2_-treated group.

As shown in Figure 3, NO.20 prevented apoptosis in H_2_O_2_-treated SH-SY5Y cells. The cytochrome c levels were higher in cells treated with H_2_O_2_ alone than in control cells, and NO.20 pretreatment abrogated the H_2_O_2_-induced cytochrome c release to the cytosol (Figure 3(a)). Thus, NO.20 maintained the mitochondrial cytochrome c levels (Figure 3(b)). Furthermore, NO.20 pretreatment suppressed H_2_O_2_-induced apoptosis by blocking the decrease in Bax (Figure 3(c)) and the increase in Bcl-2 (Figure 3(d)).

**Figure 4:**
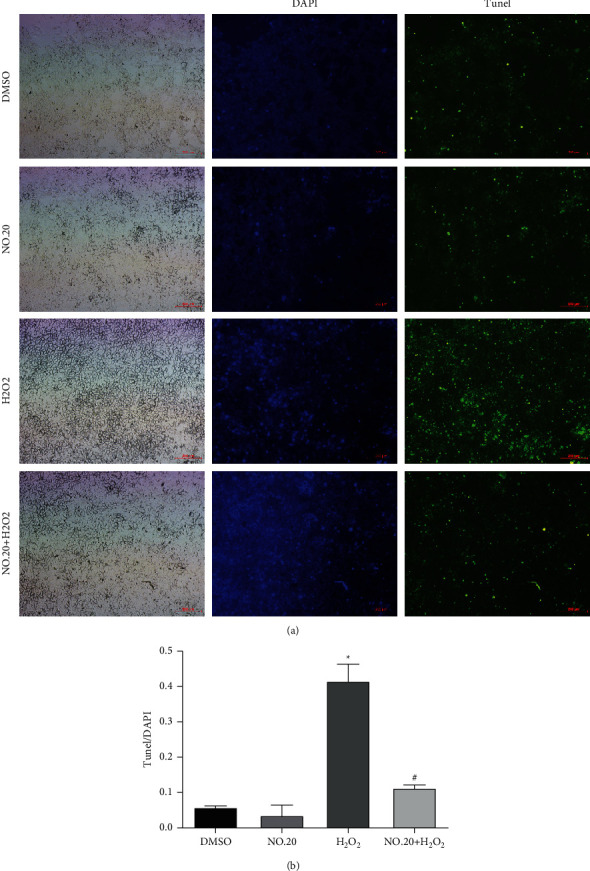
Antiapoptosis effects of NO.20 pretreatment on SH-SY5Y cells detected by the TUNEL staining test. (a) Apoptotic cells appear in green (200× magnification, scale bar = 10 *μ*m). (b) The calculated apoptosis rate. The results are presented as the mean ± SEM and represent three independent experiments. One-way ANOVA followed by Dunnett's test. ^*∗*^*P* < 0.05 vs. the DMSO group. ^#^*P* < 0.05 vs. the H_2_O_2_-treated group.

Cells treated with H_2_O_2_ alone displayed higher caspase-3 activity than control cells, and NO.20 pretreatment decreased this activity (Figure 3(e)). To confirm these results, we performed TUNEL staining and assessed the antiapoptosis effect of NO.20 (Figure 4(a)). This test showed that pretreating SH-SY5Y cells with 1 *μ*M NO.20 significantly reduced H_2_O_2_-induced apoptosis (Figure 4(b)).

### 3.2. NO.20 Elicited an Antioxidant Effect on H_2_O_2_-Treated SH-SY5Y Cells

**Figure 5:**
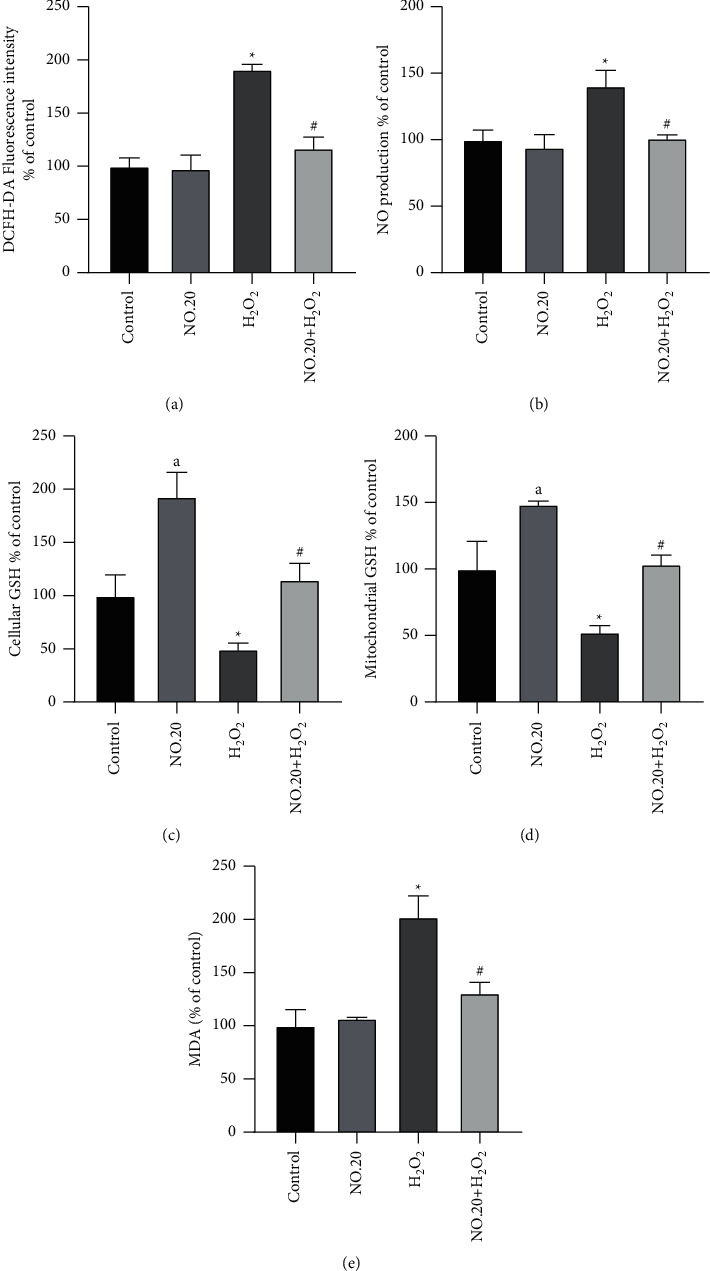
Antioxidant effects of NO.20 pretreatment on SH-SY5Y cells. (a) ROS levels. (b) NO levels. (c) Cellular GSH levels. (d) Mitochondrial GSH levels. (e) MDA levels. The results are presented as the mean ± SEM and represent three independent experiments. One-way ANOVA followed by Dunnett's test. ^*∗*^*P* < 0.05 vs. the DMSO group. ^#^*P* < 0.05 vs. the H_2_O_2_-treated group. ^*a*^*P* < 0.05 vs. the DMSO group.

Next, we examined whether NO.20 had an antioxidant effect on H_2_O_2_-treated SH-SY5Y cells. Treating SH-SY5Y cells with H_2_O_2_ elevated their ROS levels. However, NO.20 pretreatment effectively prevented this ROS level elevation (Figure 5(a)). Additionally, NO.20 significantly reduced NO production in H_2_O_2_-treated SH-SY5Y cells (Figure 5(b)). We then quantified cellular and mitochondrial GSH levels. NO.20 prevented the H_2_O_2_-induced decrease in GSH levels (Figure 5(c)), while increasing mitochondrial GSH levels (Figure 5(d)). Consequently, NO.20 abrogated the H_2_O_2_-induced MDA level increase (Figure 5(e)).

### 3.3. NO.20 Protected H_2_O_2_-Treated SH-SY5Y Cells through the Nrf2/HO-1 Pathway

**Figure 6:**
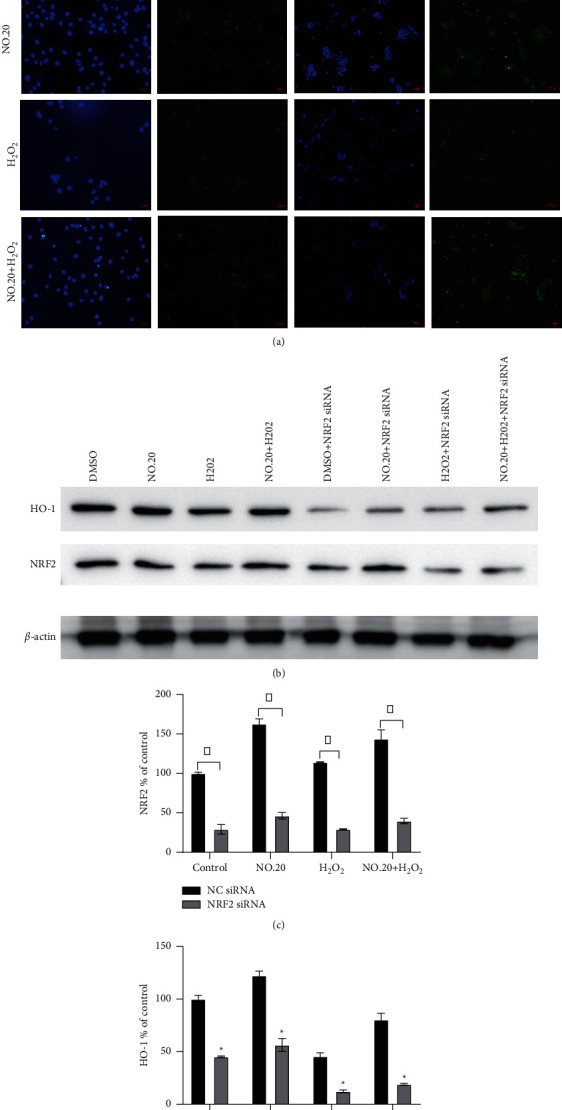
Effects of NO.20 pretreatment on Nrf2/HO-1 pathway-related gene expression. (a) Immunofluorescent staining for Nrf2 nuclear translocation with or without Nrf2 siRNA transfection. Nrf2 appears in green (200× magnification, scale bar = 10 *μ*M). (b) Relative protein expression of components of the Nrf2/HO-1 pathway in SH-SY5Y cells. (c) Quantification of Nrf2 in the nucleus with or without Nrf2 siRNA transfection. (d) Quantification of HO-1 in the nucleus with or without Nrf2 siRNA transfection. The groups were compared by two-way ANOVA analysis, followed by *t*-tests between group pairs. Data are presented as means ± SEM (*n* = 3). ^*∗*^*P* < 0.05 vs. the NC siRNA group.

To further investigate the mechanism by which NO.20 protected H_2_O_2_-treated cells, we transfected SH-SY5Y cells with Nrf2 siRNA 24 h before NO.20 treatment in the present experimental model. We then quantified nuclear Nrf2 accumulation by Western blotting. The immunofluorescence staining showed that under normal conditions, Nrf2 was located in the cytoplasm, while NO.20-stimulated cells had significantly higher nuclear Nrf2 levels. Thus, NO.20 could promote the nuclear transfer of Nrf2 (Figure 6(a)). The Western blot also showed that siRNA transfection prevented the nuclear translocation of Nrf2 in cells pretreated with NO.20 (Figures 6(b) and 6(c)). Moreover, we found that NO.20 increased HO-1 expression, while knocking down Nrf2 markedly decreased it (Figures 6(b) and 6(d)).

**Figure 7:**
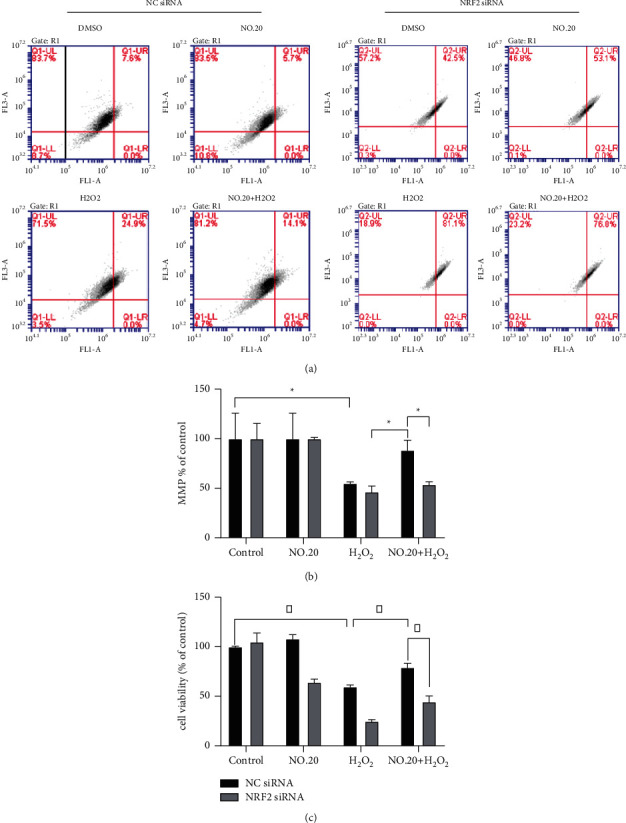
Effects of Nrf2 siRNA (24 h) on the apoptosis of SH-SY5Y cells treated or without NO.20 and/or H_2_O_2_. (a) The percentage of apoptotic SH-SY5Y cells as detected by flow cytometry. (b) MMP levels. (c) Cell viability of SH-SY5Y cells. The groups were compared by two-way ANOVA analysis, followed by *t*-tests between group pairs. Data are presented as means ± SEM (*n* = 3). ^*∗*^*P* < 0.05.

In summary, these findings confirm that NO.20 activates Nrf2, which upregulates HO-1. Nrf2 silencing also blocked the effects of NO.20 on MMP in H_2_O_2_-treated SH-SY5Y cells (Figures 7(a) and 7(b)). Finally, we found that NO.20 failed to improve the cell viability in H_2_O_2_-treated SH-SY5Y cells when Nrf2 was silenced (Figure 7(c)).

## 4. Discussion

The present study demonstrates that NO.20 exerts major neuroprotective effects in SH-SY5Y cells through Nrf2. AD is a complex pathological process. As the most critical injury mechanism, oxidative stress is a crucial target in the treatment of such diseases [21, 22]. As one of the main ROS, H_2_O_2_ is produced during the redox process and is a messenger in intracellular signaling cascades; it can penetrate the cell membrane and react with various biological targets, such as DNA, lipids, and proteins, causing nerve cell damage and even death [23]. Therefore, it has been widely used in oxidative stress-induced apoptosis models [24].

Mitochondria are closely related to apoptosis, and the depolarization of mitochondrial membrane potential is one of the earliest events in the apoptosis reaction cascade [25]. Once the mitochondrial membrane potential collapses, apoptosis is irreversible [26]. This process involves numerous genes and proteins [27]. Thus, the protective mechanism of the mitochondrial pathway could be clarified by detecting changes in ROS, NO, and GSH levels, MMP, and cytochrome c release in SH-SY5Y cells. In the present work, we found that pretreating SH-SY5Y cells with NO.20 before exposure to H_2_O_2_ prevented mitochondrial dysfunction, cell viability loss, and apoptosis. Our study has shown that H_2_O_2_ significantly increases ROS levels and damages SH-SY5Y cells. Furthermore, we found that NO.20 pretreatment in SH-SY5Y cells markedly reduced the H_2_O_2_-induced cell viability loss, ROS production, and NO and MDA levels. NO.20 also upregulated mitochondrial GSH and reduced MDA levels. Finally, it reduced the release of cytochrome c from mitochondria and prevented MMP depolarization. These results reveal that NO.20 exerts its antioxidant protective effect against H_2_O_2_-induced neurotoxicity by maintaining the stability of the mitochondrial membrane through the mitochondrial apoptotic pathway.

Bcl-2, a member of the Bcl-2 family, is the most important apoptosis suppressor gene in vivo [28]. Thus, increasing Bcl-2 expression can improve the resistance of almost all cells to apoptosis signals [29]. Bax is another proapoptotic Bcl-2 family member. Therefore, the Bax/Bcl-2 ratio plays a key role in cell proliferation and apoptosis regulation. We showed that H_2_O_2_ downregulated Bcl-2 and upregulated Bax, increasing the Bax/Bcl-2 ratio. In addition, NO.20 pretreatment prevented this effect, suggesting that NO.20 can regulate the apoptosis of SH-SY5Y cells and protect them by coordinating the expression of Bax and Bcl-2. H_2_O_2_ stimulates and activates the upstream caspases in the apoptotic pathway, then activating caspase-3 [30]. As an apoptotic executor, caspase-3 activates DNA cleavage factors, and activated endonucleases then cleave nuclear DNA, leading to cell death. In this study, NO.20 significantly reduced H_2_O_2_-induced caspase-3 expression, suggesting that NO.20 exerts its protective effect on H_2_O_2_-induced SH-SY5Y cell damage by inhibiting caspase-3 expression. Overall, these results suggest that NO.20 pretreatment prevented H_2_O_2_-induced mitochondrial dysfunction and apoptosis in SH-SY5Y cells.

ROS can activate many transcription factors, including Nrf2 [31]. Nrf2 is an essential nuclear transcription factor in the body's antioxidant stress pathway; it plays a crucial role in activating the expression of various genes related to cell protection and detoxification and plays a vital role in cell defense [32]. Notably, the Nrf2/ARE signaling pathway is a common molecular target for natural products. Under normal conditions, Nrf2 forms a covalent complex with Keap1 through an intermolecular disulfide bond, and Keap1 maintains Nrf2 in the cytoplasm. Oxidation or modification of this specific Keap1 cysteine releases Nrf2, which travels from the cytoplasm to the nucleus and reacts with antioxidant response elements. This process can activate the expression of antioxidant genes, activate the transcription of phase II detoxification enzymes and antioxidant enzyme-related genes, regulate numerous downstream molecules, induce the expression of a variety of antioxidant substances—including HO-1—and participate in the regulation of the oxidative stress response [33]. Thus, HO-1 induction is an important target of oxidative stress drugs.

We next investigated whether the antioxidant activities of NO.20 could be related to its ability to induce HO-1 expression. NO.20 pretreatment resulted in the nuclear translocation of Nrf2 and increased HO-1 protein expression, suggesting that HO-1 expression depends on Nrf2 activation. To identify the signaling pathways by which NO.20 activates Nrf2 and induces HO-1 expression, we knocked down Nrf2. We confirmed that NO.20 activated Nrf2 and induced HO-1 expression. Furthermore, we found that NO.20 pretreatment induced Nrf2 translocation into the nucleus and increased HO-1 expression. Knocking down Nrf2 abrogated these effects. Therefore, we postulate that the antioxidant and neuroprotective activities of NO.20 are largely dependent on HO-1 induction. Finally, NO.20 displayed a beneficial neuroprotective effect in an H_2_O_2_-induced oxidative stress damage in vitro model.

In conclusion, the current study is the first to report the neuroprotective effects of the abietane diterpenoid compound NO.20 (11,12-diacetyl-carnosol) extracted from rosemary. Our results suggest that the new compound exerts its antioxidant activities through the Nrf2/HO-1 pathway. Based on the mentioned evidence, this new natural product with neuroprotective properties could help develop new drugs against AD. However, further studies are necessary to optimize the structure of the new compound, and in vivo experiments are also indispensable.

## Data Availability

The datasets used and/or analyzed during the current study are available from the corresponding author upon request.

## Abbreviations

AD: Alzheimer's disease
EGCG: Epigallocatechin gallate
GSH: Glutathione
HO-1: Heme oxygenase-1
MDA: Malondialdehyde
MMP: Mitochondrial membrane potential
NO.20: 11,12-Diacetyl-carnosol
Nrf2: Nuclear factor-erythroid 2-related factor 2
ROS: Reactive oxygen species
NC: Negative control.
